# Supplementary material for: The regulatory effect of blood group on ferritin levels in aging: a retrospective study
Source: PeerJ. 2025 Apr 14;13:e19281. doi: 10.7717/peerj.19281 (PMC12005183; doi:10.7717/peerj.19281)
Supplement: Supplemental Information 2 [file peerj-13-19281-s002.docx]

**Supplementary Material**

| **Supplementary Table 1. Summary of full names and abbreviations** | | | |
| --- | --- | --- | --- |
| **Trait category** | **Abbreviation** | **Full name** | **Unit** |
|  | Fer | Ferritin | ng/ml |
| Liver function | ALT | Alanine aminotransferase | U/L |
|  | AST | Aspartate aminotransferase | U/L |
|  | Cr | Creatinine | µmol/L |
|  | GGT | Gamma-glutamyltranspeptidase | U/L |
|  | ALP | Alkaline phosphatase | U/L |
|  | GLOB | Globulin | g/L |
|  | A/G | Albumin/Globulin |  |
| Glycometabolism | Glu | Glucose | mmol/L |
| Lipid metabolism | HDL-C | High density lipoprotein cholesterol | mmol/L |
|  | LDL-C | Low density lipoprotein cholesterol | mmol/L |
|  | sd LDL-C | Small and dense low density cholesterol | mmol/L |
|  | Lpa | Lipoprotein a | mg/L |
|  | APOA1 | Apolipoprotein A1 | g/L |
| Myocardial function | LDH | Lactate dehydrogenase | U/L |
|  | LDH-1 | Lactate dehydrogenase isoenzyme | U/L |
|  | CK | Creatine kinase | U/L |
|  | CK-MB | Creatine kinase-MB isoenzyme activity | ng/ml |
|  | α-HBDH | Alpha hydroxybutyrate dehydrogenase | U/L |
|  | NT-pro BNP | N-terminal -B type natriuretic peptide precursor | pg/ml |
|  | TnT | Troponin T | µg/L |
|  | Mb | Myohemoglobin | ng/mL |
| Thyroid function | TSH | Thyroid stimulating hormone | mU/L |
|  | FT3 | Free triiodothyronine | pg/ml |
|  | T3 | Triiodothyronine | ng/ml |
|  | FT4 | Free thyroxine | pmol/L |
| Inflammatory factor | IL-6 | Interleukin-6 | pg/ml |

| **Supplementary Table 2. Normality test for continuous variables** | | | | | | | | | |
| --- | --- | --- | --- | --- | --- | --- | --- | --- | --- |
| **Variables** | **N** | **Mean** | **Standard deviation** | **Skewness** | **Kurtosis** | **Kolmogorov-Smirnov test** | | **Shapiro-Wilk test** | |
|  |  |  |  |  |  | **Statistic D value** | **p-value** | **Statistic W value** | **p-value** |
| Age | 2960 | 53.959 | 12.185 | 1.171 | 1.029 | 0.126 | p<0.001 | 0.893 | p<0.001 |
| Fer | 2960 | 288.162 | 99.894 | 2.464 | 32.782 | 0.424 | p<0.001 | 0.478 | p<0.001 |
| ALT | 2960 | 22.262 | 16.081 | 3.751 | 23.515 | 0.221 | p<0.001 | 0.696 | p<0.001 |
| AST | 2960 | 22.375 | 13.315 | 22.487 | 851.676 | 0.241 | p<0.001 | 0.399 | p<0.001 |
| ALP | 2960 | 73.943 | 25.553 | 17.373 | 618.049 | 0.288 | p<0.001 | 0.487 | p<0.001 |
| Cr | 2960 | 68.404 | 22.250 | 5.671 | 54.686 | 0.180 | p<0.001 | 0.630 | p<0.001 |
| GGT | 2960 | 30.733 | 28.747 | 4.905 | 38.736 | 0.225 | p<0.001 | 0.594 | p<0.001 |
| GLOB | 2960 | 26.969 | 3.153 | 0.245 | 1.895 | 0.228 | p<0.001 | 0.893 | p<0.001 |
| A/G | 2960 | 1.575 | 0.195 | 0.421 | 2.447 | 0.236 | p<0.001 | 0.881 | p<0.001 |
| Glu | 2960 | 5.321 | 0.732 | 1.781 | 7.202 | 0.178 | p<0.001 | 0.868 | p<0.001 |
| LDL-C | 2960 | 2.892 | 0.410 | 0.108 | 3.783 | 0.329 | p<0.001 | 0.750 | p<0.001 |
| HDL-C | 2960 | 1.233 | 0.166 | 0.302 | 3.692 | 0.339 | p<0.001 | 0.744 | p<0.001 |
| sd LDL-C | 2960 | 0.848 | 0.284 | 2.175 | 25.015 | 0.224 | p<0.001 | 0.862 | p<0.001 |
| Lpa | 2960 | 205.518 | 117.043 | 1.380 | 4.386 | 0.292 | p<0.001 | 0.797 | p<0.001 |
| APOA1 | 2960 | 1.319 | 0.194 | 0.286 | 3.174 | 0.279 | p<0.001 | 0.835 | p<0.001 |
| LDH | 2960 | 234.456 | 41.973 | -0.698 | 4.692 | 0.434 | p<0.001 | 0.620 | p<0.001 |
| LDH-1 | 2960 | 40.484 | 6.914 | -0.956 | 4.493 | 0.431 | p<0.001 | 0.619 | p<0.001 |
| CK | 2960 | 112.398 | 38.566 | 3.246 | 33.283 | 0.372 | p<0.001 | 0.545 | p<0.001 |
| CK-MB | 2960 | 15.736 | 4.454 | 0.950 | 19.046 | 0.416 | p<0.001 | 0.591 | p<0.001 |
| α-HBDH | 2960 | 168.106 | 25.968 | -0.244 | 9.172 | 0.418 | p<0.001 | 0.615 | p<0.001 |
| NT-pro BNP | 2960 | 1157.057 | 532.537 | 2.619 | 42.281 | 0.465 | p<0.001 | 0.409 | p<0.001 |
| TnT | 2960 | 0.045 | 0.031 | 32.818 | 1507.630 | 0.425 | p<0.001 | 0.175 | p<0.001 |
| Mb | 2960 | 91.523 | 57.111 | 22.611 | 762.387 | 0.425 | p<0.001 | 0.206 | p<0.001 |
| TSH | 2960 | 2.647 | 0.753 | 2.211 | 29.817 | 0.382 | p<0.001 | 0.565 | p<0.001 |
| FT3 | 2960 | 4.301 | 0.385 | 0.253 | 10.318 | 0.400 | p<0.001 | 0.581 | p<0.001 |
| T3 | 2960 | 1.510 | 0.158 | 0.713 | 9.330 | 0.408 | p<0.001 | 0.587 | p<0.001 |
| FT4 | 2960 | 15.849 | 1.101 | 0.160 | 8.003 | 0.382 | p<0.001 | 0.611 | p<0.001 |
| IL-6 | 2960 | 76.228 | 20.823 | -1.508 | 16.586 | 0.516 | p<0.001 | 0.335 | p<0.001 |

Note: N, number; Fer, ferritin; ALT, alanine aminotransferase; AST, aspartate aminotransferase; Cr, creatinine; GGT, gamma-glutamyltranspeptidase; ALP, alkaline phosphatase; GLOB, globulin; A/G, albumin/hlobulin; Glu, glucose; HDL-C, high density lipoprotein cholesterol; LDL-C, low density lipoprotein cholesterol; sd LDL-C, small and dense low density cholesterol; Lpa, lipoprotein a; APOA1, apolipoprotein A1; LDH, lactate dehydrogenase; LDH-1, lactate dehydrogenase isoenzyme; CK, creatine kinase; CK-MB , creatine kinase-MB isoenzyme activity; α-HBDH, alpha hydroxybutyrate dehydrogenase; NT-pro BNP, N-terminal -B type natriuretic peptide precursor; TnT, troponin T; Mb, Myohemoglobin; TSH, thyroid stimulating hormone; FT3, free triiodothyronine; T3, triiodothyronine; FT4, free thyroxine; IL-6, interleukin-6.
