# Supplementary material for: The regulatory effect of blood group on ferritin levels in aging: a retrospective study
Source: PeerJ. 2025 Apr 14;13:e19281. doi: 10.7717/peerj.19281 (PMC12005183; doi:10.7717/peerj.19281)
Supplement: Supplemental Information 3 [file peerj-13-19281-s003.docx]

**A Codebook of Raw Data**

Since the dataset contains categorical data that are recorded numerically, we provide a codebook to convert numbers to their respective factors to indicate meaning.

| **Title** | **Number** | **Tags** |
| --- | --- | --- |
| gender | 1 | male |
|  | 2 | female |
| blood type | 0 | O |
|  | 1 | A |
|  | 2 | B |
|  | 3 | AB |
